# Modern Sociology

**Published:** 1900-03-03

**Authors:** 


### Modern Sociology.

### THE FUTURE OF THE PEAT-BOG.

As the world grows old it becomes economical, and begins to use up materials which in its reckless youth it regarded as useless. The utilisation of waste products is one of the most conspicuous features of modern industry. Of late attention has been turned to the potentialities of peat. Everyone knew, of course, that black peat made a good fuel, which is still largely used in Ireland, Scotland, and some parts of Holland. But of the lighter coloured and more porous kind of peat no use was made until comparatively recently.

The first use of peat was as litter in stables and kennels. It is probable that in the first case its chief recommendation was cheapness. But it proved to be particularly well adapted for the purpose. It was soft, flexible, and elastic; the animal felt it to be comfortable. It was also porous, so that it retained urine, and did not become messy, and thus irritating to the skin. Dogs whose kennels were covered with peat and straw have been found to escape largely from eczema and similar diseases. It was noticed, moreover, that the offensive smell of excreta was much modified by the employment of peat, and this suggested further uses, which, however, demanded that the fibre should be further softened, and woven into a cloth. A French firm has been successful in doing this, and has produced a fabric, the generic name of which is "petanelle," and which is woven in various degrees of fineness for different purposes.

In view of the quality of peat already mentioned, that, namely, of preventing the escape of offensive odours, the peat fabrics are useful for infants' napkins and bedding. It is claimed for napkins made of this material that this combination of extreme porousness with aseptic quality prevent the effluvia from irritating the skin and causing soreness. Drawsheets of peat-fibre are also convenient for use with invalids. In a minor degree it is important that all underclothing should possess this absorbent and aseptic quality. This is claimed for peat flannel, or "flannelle Alvéolée," which contains 50 per cent. of peat fibre. This may be used in place of all knitted or woollen materials for jerseys, sweaters, and the like, as well as for underwear. It is claimed for it that peat flannel permits the complete evaporation of sweat and all particles of organic waste that are cast off by the skin, while, being aseptic, or even antiseptic in its nature, it arrests the development of malignant micro-organisms which might obtain an entrance to pores opened by exercise. The offensive odours that hang about woollen clothing after it has been for some time in use are said to be absent from this peat flannel. It is said also that it does not shrink or lose its porosity through wear or washing, and can even be immersed in antiseptic solutions, if necessary, without shrinkage or alteration.

The latest use of peat is in surgical dressings. As an absorbent of exceptional power it draws away fluid exudations from the wound, and thus tends to prevent suppuration. As it absorbs gases also, it deodorizes the infected region, while its antiseptic properties make it possible to leave a peat dressing on for a longer time than would be possible in the case of ordinary absorbent cotton. It is said, indeed, that a peat wool dressing need not be renewed oftener than every 14 or 15 days, and might even be left on for a month or more in cases of emergency, and that this gives it an advantage for country and army surgery; all of which it will be well to accept *cum grano*, as it must depend on the condition of the wound. Till lately, however, the peat dressing has had the disadvantage of being produced only in the form of wool, which had a tendency to leave broken fibres in the wound. Now, however, the peat is prepared in the form of gauze, and the former reproach is no longer justified. Peat gauze bandages are rapidly growing in popularity, as they are comfortable as well as convenient.

The value of peat in these more elaborate forms is not yet popularly known, but in the simpler form of peat powder, it is extensively used. The town of Gothenburg purchases annually 300 tons of peat from Holland, for the purification of its earth-closets, which there take the place of water closets. In places where the fall of the drainage is not sufficient, such closets, disinfected in some similar fashion, might well be introduced in this country. It is needless to say that the compound of peat with fæcal matter forms an excellent fertiliser, and needs less treatment in order to be used for this purpose than ordinary sewage. In slaughter-houses also peat powder has been used to mix with the blood to prevent putrefaction and decomposition. Of the value claimed for peat as preserving vines from the phylloxera and securing silkworms from diseases to which they are liable, we need not speak here, as these points are of no interest to us.

But to whatever uses it may be put finally, the fact seems to be established that peat has a great future before it. Even used as bedding for horses and dogs it has a distinct value. It is in itself useless to the farmer, who will sell it readily, even though when enriched by animal excreta he buys it back again as manure. As a powder it can be used in the same way as any other disinfectant agent, and as a fabric it can be employed for bandages and pads, as well as for under and even upper clothing. In short, a product which has till lately been absolutely useless has now a distinct commercial value, which is likely to increase in the near future. The scientific utilisation of peat has, as has been said, been largely carried out in France; but a company now exploits it in England. The raw material is plentiful enough as yet. Whether it may remain as cheap when landlords find out that what was waste ground can be turned to such good account is another matter. When the peat-moss blossoms out in the golden bloom of coin of the realm it will be a joyous day for many a man. He will no longer look out on the desolate waste with desponding eyes, but will see in ground for which no tenant would give him even the meanest rent a substantial source of income. This may be a dream of the future, but with the utilisation of a commodity so plentiful and so cheap as peat comes something like the realisation of the miser's dream of making something out of nothing. And, from the point of view of the every-day economist, it is matter for congratulation when a substance so plentiful and so cheap becomes a commodity of solid advantage to the community.

